# Associations between Metabolic Syndrome and Obesity-Related Indices and Bone Mineral Density T-Score in Hemodialysis Patients

**DOI:** 10.3390/jpm11080775

**Published:** 2021-08-09

**Authors:** Chih-Hsuan Wung, Cheng-Yin Chung, Pei-Yu Wu, Jiun-Chi Huang, Yi-Chun Tsai, Szu-Chia Chen, Yi-Wen Chiu, Jer-Ming Chang

**Affiliations:** 1 Department of Post Baccalaureate Medicine, Kaohsiung Medical University, Kaohsiung 807, Taiwan; knash031130@hotmail.com.tw; 2 Division of Nephrology, Department of Internal Medicine, Ministry of Health and Welfare, Pingtung Hospital, Pingtung 900, Taiwan; capella913@gmail.com; 3 Division of Nephrology, Department of Internal Medicine, Kaohsiung Medical University Hospital, Kaohsiung Medical University, Kaohsiung 807, Taiwan; wpuw17@gmail.com (P.-Y.W.); karajan77@gmail.com (J.-C.H.); lidam65@yahoo.com.tw (Y.-C.T.); chiuyiwen@gmail.com (Y.-W.C.); jemich@kmu.edu.tw (J.-M.C.); 4 Department of Internal Medicine, Kaohsiung Municipal Siaogang Hospital, Kaohsiung Medical University, 482, Shan-Ming Rd., Hsiao-Kang Dist., Kaohsiung 812, Taiwan; 5 Faculty of Medicine, College of Medicine, Kaohsiung Medical University, Kaohsiung 807, Taiwan

**Keywords:** metabolic syndrome, MetS components, obesity-related index, bone mineral density

## Abstract

Previous studies have reported inconsistent results regarding the associations between metabolic syndrome (MetS) and obesity-related indices and bone mineral density (BMD). However, no previous studies have reported these associations among hemodialysis (HD) patients. The aims of this study were to investigate associations between MetS and its components and BMD T-score in HD patients and also between obesity-related indices and BMD T-score in HD patients with and without MetS. MetS was defined according to the Adult Treatment Panel III for Asians, and BMD T-score was calculated using dual-energy X-ray absorptiometry. Eight obesity-related indices were evaluated, including lipid accumulation product (LAP), visceral adiposity index (VAI), body adiposity index, conicity index (CI), body roundness index (BRI), abdominal volume index (AVI), waist-to-height ratio (WHtR), waist–hip ratio, and body mass index (BMI). One hundred and sixty-four patients undergoing HD were enrolled, and the prevalence of MetS was 61.6%. MetS was significantly associated with high lumbar spine and total hip T-scores. Regarding the MetS components, abdominal obesity and low HDL-C were significantly associated with high lumbar spine, femoral neck, and total hip T-scores; hypertriglyceridemia was significantly associated with high lumbar spine and total hip T-scores; hyperglycemia was significantly associated with a high lumbar spine T-score, whereas high blood pressure was not associated with T-score at any site. In the patients with MetS, BMI, WHtR, AVI, and BRI were significantly associated with T-score at all sites, and high CI, VAI, and LAP were also related to a high lumbar T-score. However, these indices were not associated with T-score at any site in patients without MetS. This study demonstrated positive associations between MetS and its five components and BMD T-score among HD patients. MetS, abdominal obesity, hypertriglyceridemia, and low HDL-cholesterol were associated with low risk of osteoporosis among the HD patients. Furthermore, we found that some obesity-related indices were associated with BMD T-score among HD patients with MetS but not in those without MetS. Our study highlights the importance of BMI, WHtR, AVI, and BRI in predicting the risk of osteoporosis among HD patients with MetS. In clinical practice, they can be easily calculated through simple anthropometric measurements and routine laboratory examinations and be used to quickly and conveniently assess the risk of osteoporosis among HD patients.

## 1. Introduction

Osteoporosis is characterized by bone fragility caused by a loss of bone mass and deteriorating bone micro-architecture [1]. Hip and vertebral fractures are strongly associated with reductions in bone mineral density (BMD) of the hip and spine, respectively [2]. The risk factors associated with osteoporosis can be classified as being modifiable (such as malnutrition, cigarettes smoking, and alcohol consumption), non-modifiable (such as age, gender, and ethnicity), and secondary (such as the prolonged use of medications, diabetes mellitus, and chronic kidney disease (CKD)) [3]. Patients receiving dialysis are at a higher risk of osteoporosis and fractures than the general population [4], and the prevalence of osteoporosis in this population has been reported to range from 23% to 42% [5,6]. Moreover, osteoporosis-related fractures have been associated with a higher mortality rate in patients undergoing dialysis [7]. The pathophysiology of osteoporosis in patients with end-stage renal disease is multifactorial and complex, involving the aforementioned risk factors and CKD-related bone abnormalities [8].

Metabolic syndrome (MetS) is a group of conditions including abdominal obesity, high blood pressure, hyperglycemia, and dyslipidemia. According to the World Health Organization, the definition of obesity is having a body mass index (BMI) over 30 [9]. Individuals with predominant central obesity are prone to MetS. Each component of MetS affects bone metabolism [10]. However, previous studies have reported inconsistent and controversial relationships between components of MetS and osteoporosis [11]. With regard to the components of MetS, abdominal obesity has been shown to increase the mechanical load on the body and stimulate bone accrual [12]. Hypertension-induced abnormal calcium metabolism and interactions between bone marrow and hypertension have been reported [11,13]. Hyperglycemia has been shown to decrease bone turnover and induce osteocyte apoptosis [14], and dyslipidemia can uncouple bone remodeling leading to bone resorption [15,16]. With regard to the inconsistent findings in previous studies, abdominal obesity has both been associated with osteoporosis [17] and shown to have a protective effect against bone loss and consequently a lower risk of fractures [18]. Contradictory results have also been reported between dyslipidemia and osteoporosis [19,20]. In addition, regional discrepancies between BMD and MetS have been reported. Several studies have reported positive relationships between MetS and BMD in the United States and European countries [21,22,23], while other studies in Korea have shown a negative relationship [24,25]. Moreover, contradictory reports of the association between MetS and osteoporosis have also been reported in Taiwan [26,27]. Taken together, these findings indicate that multiple factors are associated with BMD and bone health [5].

Many studies have investigated the association between MetS and obesity-related indices among different populations [28,29,30,31]. However, no previous studies have investigated the relationship between obesity-related indices and BMD among hemodialysis (HD) patients with MetS. Therefore, the aims of this study were to investigate the associations between MetS and its components and BMD T-score in HD patients, and also between obesity-related indices and BMD T-score in HD patients with or without MetS.

## 2. Subjects and Methods

### 2.1. Study Patients and Design

A total of 178 patients who had attended the dialysis clinic at a hospital in southern Taiwan for more than 3 months and undergone maintenance HD thrice weekly for more than 3 months were included in June 2017. Each HD session lasted for 3.5–4.5 h, with a blood flow rate of 250–300 mL/minute and dialysate flow rate of 500 mL/minute. The exclusion criteria were as follows: (1) patients who did not wish to undergo dual-energy X-ray absorptiometry (DXA) examinations (*n* = 6), (2) those with below-the-knee amputations of both legs (*n* = 3), and (3) those treated with antibiotics or hospitalized within 4 weeks before study entry (*n* = 5). Therefore, a total of 164 patients (74 females; 90 males; mean age 60.1 ± 10.6 years) were included. The Institutional Review Board of Kaohsiung Medical University Hospital approved this study (KMUH-IRB-F(I)-20150074), and all of the patients provided written informed consent before enrollment. The methods were performed following relevant guidelines. 

### 2.2. BMD and Body Composition Measurements

Body composition was assessed using a Horizon Wi DXA system (Hologic, Waltham, MA, USA). BMD was assessed at three sites: total hip, femoral neck, and lumbar spine (L2–L4). To minimize variations in measurements, one radiologic technologist performed all scans and calculations. T-scores were used for comparisons. 

### 2.3. Collection of Demographic, Medical, and Laboratory Data

Demographic (age and sex) and medical history (diabetes and hypertension) information was obtained from the patients’ medical records. Twelve-hour fasting blood samples were also obtained within 1 month of enrollment and analyzed using a COBAS Integra 400 system (Roche Diagnostics GmbH, D-68298 Mannheim, Germany). 

### 2.4. Definition of MetS

The National Cholesterol Education Program Adult Treatment Panel (NCEP-ATP) III guidelines [32] and modified criteria for Asians [33] were used to define MetS as three or more of the following five criteria: (1) abdominal obesity (waist circumference (WC) >80 cm in females or >90 cm in males); (2) hyperglycemia (a previous diagnosis of diabetes or fasting whole-blood glucose concentration ≥110 mg/dL); (3) high blood pressure (a diagnosis of hypertension, receiving treatment for hypertension, systolic blood pressure ≥130 mmHg, or diastolic blood pressure ≥85 mmHg); (4) low concentration of high-density lipoprotein cholesterol (HDL-C) (<50 mg/dL in females or <40 mg/dL in males); and (5) hypertriglyceridemia (triglyceride (TG) concentration ≥150 mg/dL).

### 2.5. Calculations of Obesity-Related Indices

The following obesity-related indices were calculated. 

BMI = body weight (BW) (kg)/body height (BH)^2^ (m).

Waist–hip ratio (WHR) = WC (cm)/hip circumference (HC) (cm).

Waist-to-height ratio (WHtR) = WC (cm)/height (cm).

Abdominal volume index (AVI) = $\frac{2\times {\left({\;\mathrm{WC}}_{\left(\mathrm{cm}\right)}\;\right)}^{2}+0.7\times {\left({\;\mathrm{WC}}_{\left(\mathrm{cm}\right)}-{\mathrm{HC}\;}_{\left(\mathrm{cm}\right)}\right)}^{2}}{1000}$ [34].

Body roundness index (BRI) = $364.2-365.5\times \sqrt{1-{\left(\frac{\frac{{\mathrm{WC}}_{\left(m\right)}}{2\pi }}{0.5\times {\;\mathrm{BH}}_{\left(m\right)}}\right)}^{2}}$ [35].

Conicity index (CI) = $\frac{{\mathrm{WC}}_{\left(m\right)}}{0.109\times \sqrt{\frac{{\mathrm{BW}}_{\left(\mathrm{kg}\right)}}{{\mathrm{BH}}_{\left(m\right)}}}}$ [36]. 

Body adiposity index (BAI) = $\frac{{\;\mathrm{Hip}\;\mathrm{circumference}}_{\left(\mathrm{cm}\right)\;}}{{\mathrm{BH}}_{\left(m\right)}{}^{3/2}}-18$ [37].

Visceral adiposity index (VAI) was calculated using the following sex-specific equations: 

VAI = $\left(\frac{{\mathrm{WC}}_{\left(\mathrm{cm}\right)}}{39.68+\left(1.88\times \;\mathrm{BMI}\right)}\right)\times \left(\frac{{\mathrm{TG}}_{\left(\mathrm{mmol}/L\right)}}{1.03}\right)\times \left(\frac{1.31}{{\mathrm{HDL}}_{\left(\mathrm{mmol}/L\right)}}\right)$ in males, and

VAI = $\left(\frac{{\mathrm{WC}}_{\left(\mathrm{cm}\right)}}{36.58+\left(1.89\times \;\mathrm{BMI}\right)}\right)\times \left(\frac{{\mathrm{TG}}_{\left(\mathrm{mmol}/L\right)}}{0.81}\right)\times \left(\frac{1.52}{{\mathrm{HDL}}_{\left(\mathrm{mmol}/L\right)}}\right)$ in females [38].

Lipid accumulation product (LAP) was also calculated using the following sex-specific equations:

LAP = $\left({\;\mathrm{WC}}_{\left(\mathrm{cm}\right)}-65\right)\times {\;\mathrm{TG}}_{\left(\mathrm{mmol}/L\right)}$ in males, and

LAP = $\left({\;\mathrm{WC}}_{\left(\mathrm{cm}\right)}-58\right)\times {\;\mathrm{TG}}_{\left(\mathrm{mmol}/L\right)}$ in females [39]. 

### 2.6. Statistical Analysis

Descriptive statistics are presented as mean ± standard deviation, percentage, or median (25th–75th percentile) for TG, HD duration, and parathyroid hormone (PTH). The chi-square test was used to examine differences between groups for categorical variables, and the independent *t* test was used for continuous variables. Associations between MetS and obesity-related indices and BMD T-score were examined using multivariable linear regression analysis. A *p* value < 0.05 was considered to be statistically significant. All statistical analyses were performed using SPSS software for Windows version 20.0 (SPSS Inc. Chicago, IL, USA). The two sample means statement (POWER Procedure using SAS version 9.4, SAS Institute, Cary, NC, USA) performs a power test involving the difference between two independent means.

## 3. Results

A total of 164 patients were enrolled (54.9% men and 45.1% women) with a mean age of 60.1 ± 10.6 years. The prevalence of MetS was 61.6%.

### 3.1. Comparisons of the Baseline Characteristics of the HD Patients with and without MetS

Table 1 shows comparisons of the characteristics of the patients with and without MetS. Compared to the patients without MetS, those with MetS tended to be older and to have higher rates of diabetes and hypertension and a lower duration of HD, In addition, the patients with MetS had a lower HDL-C level and higher BW, WC, HC, lumbar spine T-score, total hip T-score, fasting glucose, and TG. Moreover, the measured obesity-related indices (BMI, WHR, WHtR, AVI, BRI, CI, BAI, VAI, and LAP) were all higher in the patients with MetS.

We calculated a sample size of 63 for the MetS (−) group and 101 for MetS (+) with lumbar spine T-score, femoral neck T-score, or total hip T-score between the two groups having a mean difference of 1.05 (standard deviation (SD) 1.60, 1.63), 0.30 (SD 0.95, 1.28), or 0.53 (SD 1.00, 1.38), respectively, which provided 99%, 42%, or 82% power to detect such a difference with a two-sample *t* test with a two-sided type I error of 0.05.

### 3.2. Associations between MetS and Its Components and T-Score in All Study Patients

Associations between MetS and its components and T-score in all of the study patients (*n* = 164) using multivariable linear regression analysis after adjusting for age, gender, log HD duration, albumin, hemoglobin, total cholesterol, low-density lipoprotein cholesterol (LDL-C), CaXP product, and log PTH are shown in Table 2. MetS was significantly associated with a high lumbar spine T-score (unstandardized coefficient (β), 1.116; *p* < 0.001) and total hip T-score (β, 0.503; *p* = 0.021), but femoral neck T-score was not. For the MetS components, hyperglycemia (β, 0.782; *p* = 0.006), hypertriglyceridemia (β, 0.674; *p* = 0.026), low HDL-C (β, 0.838; *p* = 0.002), and abdominal obesity (β, 1.091; *p* < 0.001) were significantly associated with a high lumbar spine T-score, but high blood pressure was not. In addition, abdominal obesity (β, 0.679; *p* = 0.001) and low HDL-C (β, 0.537; *p* = 0.003) were significantly associated with a high femoral neck T-score, but hypertriglyceridemia, hyperglycemia and high blood pressure were not. Moreover, low HDL-C (β, 0.658; *p* = 0.001), hypertriglyceridemia (β, 0.502; *p* = 0.026), and abdominal obesity (β, 0.678; *p* = 0.002) were significantly associated with total hip T-score, but hyperglycemia and high blood pressure were not.

### 3.3. Associations between Obesity-Related Indices and T-Score in the Patients with MetS

Associations between obesity-related indices and T-score using multivariable linear regression analysis in the patients with MetS (*n* = 101) are shown in Table 3. The following multivariable linear regression analyses were performed for different indices as shown below:

1. Adjusted for age, sex, diabetes, hypertension, log HD duration, fasting glucose, albumin, hemoglobin, log TG, total cholesterol, HDL-C, LDL-C, CaXP product, and log PTH for BMI, WHR, WHtR, AVI, BRI, CI, and BAI.
2. Adjusted for age, sex, diabetes, hypertension, log HD duration, fasting glucose, albumin, hemoglobin, total cholesterol, LDL-C, CaXP product, and log PTH for VAI.
3. Adjusted for age, sex, diabetes, hypertension, log HD duration, fasting glucose, albumin, hemoglobin, total cholesterol, HDL-C, LDL-C, CaXP product, and log PTH for LAP.

After multivariable linear regression analysis, high values of AVI (per 1; β, 0.192; *p* = 0.001), WHtR (per 0.1; β, 0.929; *p* = 0.004), BMI (per 1 kg/m^2^; β, 0.120; *p* = 0.010), VAI (per 1; β, 0.090; *p* = 0.013), CI (per 0.1; β, 0.557; *p* = 0.037), BRI (per 1; β, 0.411; *p* = 0.005), and LAP (per 10; β, 0.148; *p* = 0.024) were significantly associated with a high lumbar spine T-score, but WHR and BAI were not. In addition, high values of BRI (per 1; β, 0.267; *p* = 0.016), WHtR (per 0.1; β, 0.605; *p* = 0.014), BMI (per 1 kg/m^2^; β, 0.109; *p* = 0.002), and AVI (per 1; β, 0.138; *p* = 0.001) were significantly associated with a high femoral neck T-score, but WHR, CI, BAI, VAI, and LAP were not. Regarding total hip T-score, high values of AVI (per 1; β, 0.119; *p* = 0.011), WHtR (per 0.1; β, 0.561; *p* = 0.037), BMI (per 1 kg/m^2^; β, 0.105; *p* = 0.007), and BRI (per 1; β, 0.245; *p* = 0.043) were significantly associated with a high total hip T-score, but WHR, CI, BAI, VAI, and LAP were not.

### 3.4. Associations between Obesity-Related Indices and T-Score in the Patients without MetS

Association between obesity-related indices and T-score using multivariable linear regression analysis in the patients without MetS (*n* = 63) are shown in Table 4. There were no significant correlations between any of the indices and T-score at any site.

## 4. Discussion

In this study, we found positive correlations between MetS and its components and BMD T-score, except for high blood pressure. In addition, in the patients with MetS, high BMI, WHtR, AVI, and BRI were associated with high lumbar spine, femoral neck, and total hip T-scores. Moreover, high CI, VAI, and LAP were also correlated with a high lumbar T-score. However, none of the studied obesity-related indices were associated with T-score at any site in the patients without MetS. 

The first important finding of this study is the positive association between MetS and BMD T-score in the HD patients. MetS affects the regulation of hormones, mechanical loading of the body, and biochemical profile, all of which affect bone health. Many studies have suggested that MetS is associated with BMD through mechanical loading and adiposity [40]. Visceral adipose tissue resulting in central obesity can produce adipokines and affect bone health. Leptin is a peptide hormone secreted by adipocytes, and it can control food intake via leptin receptors in the hypothalamus [41]. Individuals with hyperleptinemia consume more food and gain weight, leading to increased mechanical load [42]. Vaspin is a novel adipokine secreted by visceral adipose tissue, which can induce bone formation by protecting osteoblasts from apoptosis, and it has been shown to suppress bone erosion through osteoclast inhibition [43]. Omentin-1 is a novel visceral adipose-tissue-derived adipokine that can block the inflammatory responses and impair the anti-osteoblastic and pro-osteoclastic effects of activated macrophages [44]. Taken together, these findings suggest that MetS and obesity increase BMD through both mechanical load and adipokine regulation.

We also analyzed each component of MetS and the associations with T-score, and found that abdominal obesity, hypertriglyceridemia, low HDL-C, and hyperglycemia were associated with T-score, whereas high blood pressure was not. Patients undergoing chronic dialysis have a lower BMD than healthy subjects of similar age due to CKD-related mineral and bone disorders [45]. Abdominal obesity can reflect higher mechanical loading on the bone, which then stimulates bone accrual [12]. Many studies have reported a positive relationship between TG level and BMD [46,47,48]. TG levels are associated with abdominal fat mass, which is also related to abdominal obesity [49]. The association between low HDL-C and high BMD is consistent with the study by Jiang et al.; however the mechanism underlying this association is unclear due to complex genetic and molecular factors [50]. However, we did not find an association between high blood pressure and T-score. Calcium abnormalities are a key factor linking hypertension and osteoporosis. Hypertension is related to high sodium intake, which causes an increase in urinary calcium excretion and, subsequently, a decrease in calcium blood level. This in turn can result in the upregulation of the PTH level, leading to bone turnover [11,51]. In contrast to our study, Hanley et al. reported an association between hypertension and higher BMD in both genders [52]. In addition, in a study conducted by Tseng et al., there was no significant association between BMD and systolic blood pressure in either sex, and a strong inverse relationship was found between bone mineral loss and diastolic blood pressure in both sexes [53]. Moreover, in a study of women and men with hypertension, Yang et al. found lower femoral neck BMD in the women and higher femoral neck BMD in the men [54]. Further large population studies are needed to evaluate the relationship between hypertension and BMD.

The second important finding of this study is that high BMI, WHtR, AVI, and BRI were associated with high lumbar spine, femoral neck, and total hip T-scores in the patients with MetS. We also found associations between high CI, VAI, and LAP and a high lumbar T-score. Dogan et al. also reported an association between a high BMI and high BMD, consistent with our findings [55]. Several studies have reported that a high BW can lead to bone remodeling to compensate for mechanical load [56,57]. Other studies have reported associations between BMI and leptin level, leading to the production and activation of osteoblasts [58,59]. In addition, high WHtR was associated with high T-score in our study. WHtR is based on WC and reflects central obesity [60]. Abdominal obesity may increase BMD by stimulating bone growth and increasing the bones’ weight bearing capacity [61]. AVI and BRI are used to assess obesity and are associated with impaired glucose tolerance, and they have been reported to be good indicators of MetS status [34,62]. Overweight and obesity increase mechanical load. Taken together, these findings may explain the association between BMD and hyperglycemia and MetS. High CI, VAI, and LAP were also associated with a high lumbar T-score in the present study. CI reflects central obesity and has also been reported to be a good indicator of MetS status [63]. VAI is a surrogate of visceral fat accumulation, and it can also be used to assess central obesity [64]. LAP can be used to assess visceral fat and TG levels, and it has been shown to reflect the risk of MetS and cardiovascular disease [65]. The possible explanation for the associations between CI, VAI, and LAP with BMD may be related to central obesity and function of the lumbar spine, which is to bear the upper part of the body. Central obesity increases the mass of the upper body leading to direct mechanical load on the lumbar spine. This can then lead to an increase in bone remodeling and elevated BMD. 

Another important finding of this study is that there was no association between any of the obesity-related indices and BMD T-score among the HD patients without MetS. In our study patients, the duration of HD was longer in those without MetS than in those with MetS. Long-term dialysis can worsen inflammation-mediated proteolysis, hyper-metabolism, and nutrient loss, and these factors can lead to malnutrition [66]. Weight loss is often an obvious sign of malnutrition [67], which is inconsistent with the characteristics of MetS. Another possible explanation may be the small number of patients in this group. Therefore, malnutrition and small patient number may partially explain the non-significant association between the obesity-related indices and BMD in the patients without MetS. 

There are several limitations to this study. First, we enrolled the study patients from a single regional hospital in southern Taiwan, and therefore, our findings may not be generalizable to other areas. Second, due to the cross-sectional design of the study, we could not assess causal relationships and long-term clinical outcomes. Further longitudinal studies are needed to verify our findings. Third, all of the participants in our study were Taiwanese, and therefore our conclusions may not be generalizable to other ethnicities. Nonetheless, our results highlight the importance of MetS and obesity-related indices on osteoporosis in HD patients. Finally, the overall number of patients in this study was low, future large-scale research are needed.

In conclusion, we identified associations between MetS and its five components and obesity-related indices and BMD T-score among HD patients. MetS, abdominal obesity, hypertriglyceridemia, and low HDL-cholesterol were associated with low risk of osteoporosis among the HD patients. We also found that some obesity-related indices were associated with BMD T-score among the HD patients with MetS, but not in those without MetS. Our study highlights the importance of BMI, WHtR, AVI, and BRI in predicting the risk of osteoporosis among the HD patients with MetS. In clinical practice, they can be easily calculated through simple anthropometric measurements and routine laboratory examinations and be used to quickly and conveniently assess the risk of osteoporosis among HD patients.

## Figures and Tables

**Table 1:** Comparison of baseline characteristics among chronic HD patients stratified by MetS status.

Characteristics	All Patients (*n* = 164)	MetS (−) (*n* = 63)	MetS (+) (*n* = 101)	*p*
Age (years)	60.1 ± 10.6	58.9 ± 11.2	60.9 ± 10.2	0.236
Men (%)	54.9	52.4	56.4	0.612
Diabetes (%)	51.8	23.8	69.3	<0.001
Hypertension (%)	92.7	82.5	99.0	<0.001
Duration of HD (years)	6.9 (3.3–13.1)	10.5 (6.0–16.6)	4.8 (2.5–9.6)	<0.001
Body height (cm)	161.6 ± 8.2	160.9 ± 8.3	162.0 ± 8.1	0.385
Body weight (kg)	62.6 ± 12.0	56.0 ± 9.7	66.8 ± 11.6	<0.001
Waist circumference (cm)	87.2 ± 10.9	79.3 ± 10.0	92.0 ± 8.3	<0.001
Hip circumference (cm)	92.8 ± 7.6	89.2 ± 6.5	95.0 ± 7.5	<0.001
DXA Parameters				
Lumbar spine T-score	−1.21 ± 1.69	−1.89 ± 1.60	−0.84 ± 1.63	<0.001
Femoral neck T-score	−2.29 ± 1.18	−2.48 ± 0.95	−2.18 ± 1.28	0.139
Total hip T-score	−1.79 ± 1.28	−2.13 ± 1.00	−1.60 ± 1.38	0.009
Laboratory parameters				
Fasting glucose (mg/dL)	111.4 ± 43.3	90.8 ± 21.1	124.3 ± 48.4	<0.001
Albumin (g/dL)	3.9 ± 0.3	3.9 ± 0.2	3.9 ± 0.3	0.091
Hemoglobin (g/dL)	10.3 ± 1.3	10.3 ± 1.5	10.4 ± 1.4	0.689
Triglycerides (mg/dL)	111 (82.3–164.8)	81 (66–102)	146 (104.5–205)	<0.001
Total cholesterol (mg/dL)	172.2 ± 42.6	168.8 ± 35.1	174.3 ± 46.7	0.423
HDL-cholesterol (mg/dL)	44.0 ± 12.9	53.6 ± 13.2	38.0 ± 8.4	<0.001
LDL-cholesterol (mg/dL)	89.4 ± 29.6	84.4 ± 27.1	92.6 ± 30.8	0.086
CaXP product (mg^2^/dL^2^)	41.4 ± 10.1	41.3 ± 9.7	41.4 ± 10.4	0.921
PTH (pg/mL)	301.1 (159.4–507.7)	284.6 (158.4–506.4)	319.2 (159.7–513.1)	0.362
MetS component				
Abdominal obesity (%)	54.9	19.0	77.2	<0.001
Hypertriglyceridemia (%)	32.9	6.3	49.5	<0.001
Low HDL-cholesterol (%)	57.9	22.2	80.2	<0.001
Hyperglycemia (%)	61.0	30.2	80.2	<0.001
High blood pressure (%)	92.7	82.5	99.0	<0.001
Obesity-related indices				
BMI (kg/m^2^)	23.9 ± 4.0	21.6 ± 3.1	25.4 ± 3.8	<0.001
WHR	0.94 ± 0.08	0.89 ± 0.07	0.97 ± 0.06	<0.001
WHtR	0.54 ± 0.07	0.49 ± 0.06	0.57 ± 0.05	<0.001
AVI	15.5 ± 3.8	12.8 ± 3.2	17.1 ± 3.1	<0.001
BRI	4.2 ± 1.4	3.3 ± 1.2	4.8 ± 1.2	<0.001
CI	1.29 ± 0.09	1.23 ± 0.09	1.32 ± 0.07	<0.001
BAI	27.4 ± 4.6	26.0 ± 4.1	28.2 ± 4.7	0.002
VAI	6.4 ± 7.1	2.9 ± 2.2	8.4 ± 8.2	<0.001
LAP	46.6 ± 53.0	19.0 ± 14.9	63.2 ± 60.3	<0.001

Abbreviations: HD, hemodialysis; MetS, metabolic syndrome; DXA, dual-energy X-ray absorptiometry; BMD, bone mineral density; HDL; high-density lipoprotein; LDL, low-density lipoprotein; CaXP product, calcium × phosphorus product; PTH, parathyroid hormone; BMI, body mass index; WHR, waist–hip ratio; WHtR, waist-to-height ratio; AVI, abdominal volume index; BRI, body roundness index; CI, conicity index; BAI, body adiposity index; VAI, visceral adiposity index; LAP, lipid accumulation product.

**Table 2:** Association between MetS and its components and BMD T-score using multivariable linear regression analysis in all study patients (*n* = 164).

Parameters	Lumbar Spine T-Score		Femoral Neck T-Score		Total Hip T-Score	
	Coefficient β (95% CI)	*p*	Coefficient β (95% CI)	*p*	Coefficient β (95% CI)	*p*
MetS	1.116 (0.543, 1.689)	<0.001	0.374 (−0.018, 0.766)	0.061	0.503 (0.076, 0.931)	0.021
MetS component						
Abdominal obesity	1.091 (0.514, 1.668)	<0.001	0.679 (0.299, 1.058)	0.001	0.678 (0.258, 1.099)	0.002
Hypertriglyceridemia	0.674 (0.083, 1.264)	0.026	0.314 (−0.092, 0.719)	0.128	0.502 (0.061, 0.942)	0.026
Low HDL-cholesterol	0.838 (0.313, 1.363)	0.002	0.537 (0.184, 0.890)	0.003	0.658 (0.274, 1.043)	0.001
Hyperglycemia	0.782 (0.225, 1.339)	0.006	0.020 (−0.363, 0.402)	0.918	0.172 (−0.247, 0.591)	0.418
High blood pressure	0.203 (−0.907, 1.314)	0.718	−0.593 (−1.342, 0.156)	0.119	−0.456 (−1.281, 0.370)	0.277

Values expressed as unstandardized coefficient β and 95% confidence interval (CI). Abbreviations are the same as in Table 1. Adjusted for age, gender, log HD duration, albumin, hemoglobin, total cholesterol, LDL-cholesterol, CaXP product, and log PTH.

**Table 3:** Association between obesity-related indices and BMD T-score using multivariable linear regression analysis in patients with MetS (*n* = 101).

Obesity-Related Indices	Lumbar Spine T-Score		Femoral Neck T-Score		Total Hip T-Score	
	Coefficient β (95% CI)	*p*	Coefficient β (95% CI)	*p*	Coefficient β (95% CI)	*p*
BMI (per 1 kg/m^2^) *	0.120 (0.029, 0.211)	0.010	0.109 (0.041, 0.177)	0.002	0.105 (0.029, 0.181)	0.007
WHR (per 0.1) *	0.419 (−0.131, 0.968)	0.133	0.335 (−0.075, 0.745)	0.108	0.323 (−0.128, 0.773)	0.158
WHtR (per 0.1) *	0.929 (0.304, 1.555)	0.004	0.605 (0.128, 1.082)	0.014	0.561 (0.034, 1.089)	0.037
AVI (per 1) *	0.192 (0.087, 0.297)	0.001	0.138 (0.058, 0.219)	0.001	0.119 (0.028, 0.209)	0.011
BRI (per 1) *	0.411 (0.129, 0.692)	0.005	0.267 (0.052, 0.481)	0.016	0.245 (0.008, 0.483)	0.043
CI (per 0.1) *	0.557 (0.034, 1.080)	0.037	0.215 (−0.184, 0.613)	0.287	0.168 (−0.269, 0.606)	0.445
BAI (per 1) *	0.063 (−0.017, 0.143)	0.121	0.030 (−0.029, 0.090)	0.314	0.030 (−0.036, 0.095)	0.367
VAI (per 1) †	0.090 (0.019, 0.161)	0.013	0.019 (−0.034, 0.072)	0.485	0.031 (−0.027, 0.088)	0.291
LAP (per 10) #	0.148 (0.020, 0.277)	0.024	0.057 (−0.043, 0.158)	0.257	0.081 (−0.028, 0.191)	0.142

Values expressed as unstandardized coefficient β and 95% confidence interval (CI). Abbreviations are the same as in Table 1. * Adjusted for age, gender, diabetes, hypertension, log HD duration, fasting glucose, albumin, hemoglobin, log triglyceride, total cholesterol, HDL-cholesterol, LDL-cholesterol, CaXP product, and log PTH. † Adjusted for age, gender, diabetes, hypertension, log HD duration, fasting glucose, albumin, hemoglobin, total cholesterol, LDL-cholesterol, CaXP product, and log PTH. # Adjusted for age, gender, diabetes, hypertension, log HD duration, fasting glucose, albumin, hemoglobin, total cholesterol, HDL-cholesterol, LDL-cholesterol, CaXP product, and log PTH.

**Table 4:** Association between obesity-related indices and BMD T-score using multivariable linear regression analysis in patients without MetS (*n* = 63).

Obesity-Related Indices	Lumbar Spine T-Score		Femoral Neck T-Score		Total Hip T-Score	
	Coefficient β (95% CI)	*p*	Coefficient β (95% CI)	*p*	Coefficient β (95% CI)	*p*
BMI (per 1 kg/m^2^) *	0.041 (−0.131, 0.213)	0.631	0.059 (−0.031, 0.149)	0.194	0.051 (−0.047, 0.148)	0.299
WHR (per 0.1) *	0.226 (−0.626, 1.077)	0.592	0.410 (−0.060, 0.879)	0.085	0.321 (−0.173, 0.814)	0.195
WHtR (per 0.1) *	−0.032 (−0.944, 0.879)	0.943	0.134 (−0.377, 0.646)	0.597	0.089 (−0.447, 0.625)	0.738
AVI (per 1) *	0.084 (−0.082, 0.250)	0.312	0.083 (−0.010, 0.175)	0.078	0.066 (−0.031, 0.163)	0.177
BRI (per 1) *	−0.036 (−0.505, 0.432)	0.876	0.059 (−0.205, 0.323)	0.652	0.035 (−0.241, 0.312)	0.797
CI (per 0.1) *	0.215 (−0.447, 0.876)	0.513	0.115 (−0.274, 0.504)	0.553	0.061 (−0.347, 0.469)	0.764
BAI (per 1) *	−0.155 (−0.321, 0.011)	0.066	−0.065 (−0.150, 0.021)	0.136	−0.052 (−0.142, 0.037)	0.243
VAI (per 1) †	0.056 (−0.195, 0.308)	0.651	−0.044 (−0.183, 0.095)	0.525	−0.036 (−0.181, 0.110)	0.620
LAP (per 10) #	0.184 (−0.213, 0.582)	0.352	0.046 (−0.185, 0.277)	0.687	0.003 (−0.239, 0.246)	0.977

Values expressed as unstandardized coefficient β and 95% confidence interval (CI). Abbreviations are the same as in Table 1. * Adjusted for age, gender, diabetes, hypertension, log HD duration, fasting glucose, albumin, hemoglobin, log triglyceride, total cholesterol, HDL-cholesterol, LDL-cholesterol, CaXP product, and log PTH. † Adjusted for age, gender, diabetes, hypertension, log HD duration, fasting glucose, albumin, hemoglobin, total cholesterol, LDL-cholesterol, CaXP product, and log PTH. # Adjusted for age, gender, diabetes, hypertension, log HD duration, fasting glucose, albumin, hemoglobin, total cholesterol, HDL-cholesterol, LDL-cholesterol, CaXP product, and log PTH.

## Data Availability

Data may be available upon request to interested researchers. Please send data requests to: Szu-Chia Chen, PhD, MD. Division of Nephrology, Department of Internal Medicine, Kaohsiung Medical University Hospital, Kaohsiung Medical University.
